# Feasibility of the consultation-based reassurance questionnaire in Danish chiropractic practice

**DOI:** 10.1186/s12998-018-0197-8

**Published:** 2018-08-30

**Authors:** Alice Kongsted, Magnus Rudbæk Christensen, Karl Kristian Ingersen, Tue Secher Jensen

**Affiliations:** 1 Nordic Institute of Chiropractic and Clinical Biomechanics, Campusvej 55, 5230 Odense M, Denmark; 2 Department of Sports Science and Clinical Biomechanics, University of Southern Denmark, Odense M, Denmark; 3 Diagnostic Imaging, Silkeborg Regional Hospital, Silkeborg, Denmark; 4 Department of Clinical Medicine, Aarhus University, Aarhus, Denmark

**Keywords:** Back pain, Chiropractic, Patient involvement, Primary health care, Questionnaires, Reassurance

## Abstract

**Background:**

Reassuring information is recommended in clinical guidelines for the treatment of low back pain (LBP), but has not been clearly defined. The Consultation-based Reassurance Questionnaire (CRQ) was developed as a tool for measuring to what extent reassurance is present in back pain consultations and may provide important information about the clinical encounter. Until now the CRQ has only been tested in general practice patients in the UK although many patients with LBP are seen outside of this setting. The objectives of this study were to translate the CRQ into Danish, test its feasibility in chiropractic practice, and determine if CRQ scores were associated with satisfaction with care and perceived pain control.

**Methods:**

On the day of the first visit for a LBP episode, patients received an electronic survey including the CRQ. Distributions and completeness of responses on the four subscales of the CRQ (data-gathering, relationship-building, generic reassurance, cognitive reassurance) were assessed, and internal consistency for each subscale calculated as Cronbach’s alpha. Outcomes at 2 weeks were; satisfaction with care (5-point Likert scale dichotomised into yes/no) and ability to control pain (0–10). Associations of the CRQ with patient characteristics and outcomes were determined in mixed models to account for dependency of observations within clinics.

**Results:**

From 964 patients visiting between November 2016 and October 2017 with new episodes of LBP, 717 completed the CRQ with no more than 1% missing values on any single item. The internal consistency was acceptable for all subscales (0.67–0.86). Scores were generally high, and more so in patients visiting a chiropractor for the first time. All four subscales were positively associated with satisfaction (Odds ratios 1.08–1.23) and generic reassurance was weakly associated with pain control (β = 0.07 [95% CI 0.03–0.11]).

**Conclusions:**

The CRQ was feasible for use in a Danish chiropractic setting and scores on all four reassurance subscales related positively to patients’ satisfaction. Patients who had visited a chiropractor previously reported slightly lower levels of reassuring information, and it should be explored if this is in accordance with the patients’ needs. The potential impact on patient outcomes needs investigation.

## Background

Back pain is the most frequent cause for care seeking in parts of the world and globally the leading cause of disability [1, 2]. In most people, it is a recurrent condition and although the prognosis of single episodes is good, many people live with some degree of back pain for extended periods of time [3]. As there is no permanent cure for back pain, care should first and foremost enable patients to self-manage their pain episodes [4, 5] and care should be taken that clinicians do not induce pain related fear or negative expectations of the future with potential negative effects on patients’ outcome [6, 7].

Because the support of positive health beliefs and self-efficacy seem central to people with back pain, clinical guidelines generally agree that people seeking care for back pain should have reassuring information as part of the consultation to help people understand the nature of back pain [4]. Still, patients with persistent back pain and fear of movement often cannot make sense of their pain and many ascribe the pain to structural damage [8], or think of their body as a ‘broken machine’ [9]. In one study, 89% of the participating patients claimed these beliefs to be adopted from health care providers [9]. Thus, clearly reassuring messages are not always effectively conveyed, but until recently there has not been a standardised way to capture to what extent reassurance is part of back pain care.

The Consultation-based Reassurance Questionnaire (CRQ), published in 2016, was developed as a tool for measuring to what extent back pain consultations include aspects of affective and cognitive reassurance and how patients perceive the way information is gathered during the consultation [10]. The CRQ includes 12 items covering four subscales of data-gathering, relationship-building, generic reassurance, and cognitive reassurance. The questionnaire was tested in two samples from general practice in the UK and demonstrated acceptable reliability and high item-total correlations within subscales. The evidence for associations between the CRQ and patient outcomes is up to now very sparse, but suggests that the CRQ subscales may be positively correlated with patient satisfaction, patient enablement and pain reduction [10, 11].

The CRQ provides a tool for quantitative investigations of reassurance, an option for gaining insights in the impact of reassuring information on patient outcomes, and potentially for evaluating ways of educating clinicians in providing reassurance. Thus, there are important potential benefits of the CRQ, but till now it has only been tested in general practice patients in the UK. The objective of this study was to translate the CRQ into Danish and test its feasibility in chiropractic patients. We specifically aimed at determining the completeness and distribution of scores and the construct validity of the subscales. Further, to explore if age, gender, educational level, symptom duration, or previous chiropractic care were associated with reassurance scores. Lastly, we investigated if CRQ scores were associated with satisfaction with care and with the patients’ self-perceived control of pain after 2 weeks, and whether this association differed between patients who had previously consulted a chiropractor and those who had not.

## Method

The CRQ was translated from English to Danish and incorporated in the Danish Chiropractic back pain Cohort (ChiCo). This study uses data from ChiCo from the period November 1 2016 to October 31 2017. Patients completed surveys including the CRQ on the day they consulted the chiropractor for low back pain (LBP) and reported satisfaction with care and perceived ability to control pain at a follow up 2 weeks later. Data were collected using the electronic data capture software REDCap licensed by Odense Patient data Explorative Network (OPEN).

### Translation of the CRQ

The translation of the CRQ was conducted as recommended by forward and back translation [12]. The forward translation was performed by two persons with Danish as their mother tongue: A back pain researcher who is familiar with English as working language and a layperson who has a master’s degree in English Literature. After the independent translations of the questionnaire, the translations were compared and a common version agreed on. The back translation was performed by two persons who are native English speakers and have lived in Denmark and used Danish for more than 10 years. One is a back-pain researcher and one is a layperson. The wording of the back translated version was compared to the original CRQ and two of the authors (AK and TSJ) decided on the final version.

### Setting

Four private chiropractic clinics with a total of 18 chiropractors recruited study participants. The clinics that were asked to take part in the ChiCo were in the Central Denmark Region and chosen among clinics that had a digital radiography system, which was required for subprojects related to imaging. In Denmark, chiropractors are self-employed and have a contract with the Board of Wages and Fees that regulates costs for care. Approximately 20% of payment for chiropractic services is reimbursed by national health insurance. Patients seek care from chiropractors without requirement of a referral.

### Participants

Patients initiating care (not visiting for a follow up consultation) for non-specific LBP or LBP with radiculopathy were eligible for inclusion if above 18 years, Danish speaking, and having access to an email account. Patients were not included if immediate referral for surgery was required or if LBP was suspected to be caused by systemic pathology. This would also mean exclusion if occurring after study participation had started.

### Data collection

On the day of the initial visit to the chiropractor the patients completed the first part of the baseline questionnaire in the reception area before seeing the chiropractor and a second part of the baseline questionnaire was received electronically directly after the consultation via a link to their email address. Within a few days after inclusion a research assistant called the participants to welcome them to the study, answer questions about participation, and remind them to complete the second part of the baseline questionnaire if that had not been done.

#### The consultation-based reassurance questionnaire

Consultation reassurance was measured in the second part of the baseline on the CRQ (subscales data-gathering (items 6, 9, 11), relationship-building (items 2, 8, 10), generic reassurance (items 1, 3, 5), and cognitive reassurance (items 4, 7, 12)) [10]. For each subscale there are three items, each answered by indicating “to what extent did the chiropractor…” e.g. “tell you that you should not be worried” (0 = not at all; 6 = a great deal) resulting in sum scores for each subscale ranging from 0 (no reassurance) to 18 (highest extent of reassurance).

#### Additional baseline information

From the first part of the baseline questionnaire: Age and gender (personal identification number); LBP intensity (Numeric Rating Scale 0–10) [13]; leg pain intensity (Numeric Rating Scale 0–10); episode duration (1–2 days, 3–7 days, 1–2 weeks, 2–4 weeks, 1–3 months, 3–12 months, > 12 months); and pain control (Örebro Musculoskeletal Pain Questionnaire (ÖMPQ) 0 = Can’t control at all, 10 = Can control it completely) [14].

From the second part of the baseline questionnaire: Education (no qualification, high school, vocational training, higher education 2–3 yr., higher education 3–4 yr., higher education > 4 yr); and previous chiropractic care (yes/no).

#### Two-week outcomes

Satisfaction with care was defined as 4 or 5 on a six point Likert scale “All in all are you satisfied with the chiropractor’s care?” (0 = Not at all; 5 = To a very high degree), and pain control (0 = Can’t control at all, 10 = Can control it completely).

### Analyses

Patient characteristics were described as medians with interquartile range (IQR) or proportions. The degree of missing values on the CRQ was reported as proportions missing on each item in people who started filling in the survey.

Before conducting additional analyses we dropped observations where more than 6 of 12 items were missing on the CRQ. Other missing items were imputed using chained multiple imputations based on all baseline variables including the CRQ items and extracting one of five imputed datasets for the analyses. The distributions of scores were illustrated in histograms for the subscales and the floor and ceiling described as the proportion of patients scoring in the extremes of each item and subscale. The internal consistency of the four subscales were quantified by Cronbach’s alpha. Associations between the CRQ subscales and age (categorised as < 35, 35–50, > 50); sex; educational level; symptom duration (< 1 month, 1–3 months, > 3 months); and previous visit to a chiropractor were tested in linear mixed models with CRQ subscales as the dependent variable and a random intercept for clinics to account for dependency of observations. Mixed models were also used to investigate if CRQ subscales were associated with the outcomes satisfaction and pain control at 2-weeks follow up. Pain control at baseline was included as a covariate in the analysis of that outcome. Potential effect moderation by previous chiropractic care was tested by adding an interaction between previous care and the CRQ scale to the model. All analyses were performed using Stata/MP 15.1 (StataCorp LLC, TX 77845, USA).

## Results

### Translation

The translators agreed to a high degree in both the forward and the back translation, and the back translated version resembled the original version very well. The most substantial differences were: In item 6 ‘while you were talking’ was back translated as ‘while you spoke’, in item 8 ‘put you at ease’ was back translated as ‘calmed you down’ and as ‘reassured you’, and in item 9: ‘what you had told them’ was back translated as ‘what you have said’ and ‘what you had said to him/her’. These differences were not considered substantial or an indication of uncertainty in the translation. The Danish version is available from the Appendix: Table 5.

### Study sample

**Fig. 1:**
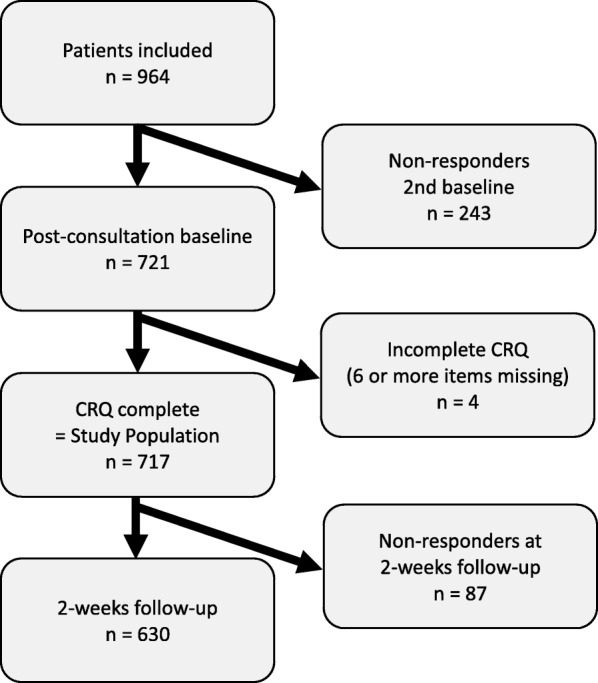
Study flow-chart

A total of 964 patients were included, 721 (75%) completed the second part of the baseline questionnaire and 717 of these, who responded to six or more questions of the CRQ, composed the study sample. The 2-week follow up was completed by 630 (88%) of the study sample (Fig. 1). Characteristics of the study population compared to non-responders are summarised in Table 1. There was a higher proportion of males among those who did not complete the second part of the baseline as compared to the study sample and they were slightly younger, but did not differ from the study population in terms of symptom severity or duration. Non-responders at the 2-weeks follow up were less likely to have visited a chiropractor before than responders, but did not differ substantially from the responders on other baseline characteristics or on CRQ scores (Table 1).

**Table 1:** Patient characteristics of the study sample and included patients who did not complete baseline or follow-up questionnaires

	Non-responders at baseline^a^ (*n* = 247)	Study sample (*n* = 717)	Responders 2-weeks (*n* = 630)	Non-responders 2-weeks (*n* = 87)
Females	35%	43%	43%	44%
Years of age, mean (SD)	41 (14)	46 (13)	46 (13)	41 (12)
LBP intensity, median (IQR)	7 (5–8)	7 (5–8)	7 (5–8)	7 (6–8)
Leg pain intensity, median (IQR)	2 (0–5)	2 (0–5)	2 (0–5)	2 (0–5)
Episode duration, %				
< 7 days	52%	50%	50%	50%
1–4 weeks	25%	20%	20%	17%
1–3 months	11%	11%	11%	13%
3–12 months	5%	7%	7%	5%
> 12 months	7%	11%	11%	15%
Pain control, median (IQR)	5 (4–7)	5 (4–7)	5 (4–7)	5 (4–7)
Education, %				
No qualification		15%	15%	18%
Education < 3 years	NA	43%	44%	34%
Education 3+ years		38%	37%	43%
Other		4%	4%	5%
Previous chiropractic care, %	NA	52%	54%	40%
CRQ scores, median (IQR)				
Data-gathering		14 (11–16)	14 (11–16)	14 (11–16)
Relationship-building	NA	15 (12–17)	14 (12–17)	15 (11–16)
Generic reassurance		12 (8–15)	12 (8–15)	12 (8–15)
Cognitive reassurance		14 (11–16)	14 (11–16)	13 (11–16)

^a^No response to 2nd baseline or incomplete CRQ

### Completeness and internal consistency

The CRQ items had few missing values. The largest proportion of missing values was 1% for the items “*Encourage you to voice your concerns regarding your symptoms*” and *“Put you at ease”* (Table 2). All 12 items were completed by 681 patients (95% of the study population). The internal consistency was high for the subscales relationship-building, generic reassurance, and cognitive reassurance (Cronbach’s alpha 0.78–0.86). Cronbach’s alpha for data-gathering was slightly lower (0.67) due to a lower item-test correlation for item 6 (*Listen attentively while you were talking*) of 0.67 compared to all other item-test correlations that were 0.8 or above.

**Table 2:** Completeness and distributions of scores on each item of the Consultation Reassurance Questionnaire (*n* = 717)

Subscales Items	Missing values, %	Score^a^, Median (IQR)	Floor, % minimum score	Ceiling, % max score
Subscale: Data-gathering			0.1%	14%
Listen attentively while you were talking	0.3%	5 (5–6)	0.1%	49%
Summarise what you had told her/him	0.7%	5 (3–6)	2%	25%
Encourage you to voice your concerns regarding your symptoms	1%	4 (3–5)	7%	21%
Subscale: Relationship-building			0%	18%
Show that he/she understood your concerns	0.6%	5 (4–6)	0.7%	31%
Put you at ease	1%	4 (3–5)	3%	24%
Show a genuine interest in your problem	0.4%	5 (4–6)	0.3%	43%
Subscale: Generic reassurance			2.5%	10%
Tell you that everything would be fine	0.7%	4 (2–5)	10%	15%
Reassure you that he/she had no serious concerns about your back	0.7%	4 (3–5)	5%	23%
Tell you that you should not be worried	0.3%	4 (3–5)	6%	18%
Subscale: Cognitive reassurance			0.7%	14%
Explain how the treatment offered would help with your problem	0.7%	5 (3–5)	3%	22%
Make sure you understood what your treatment plan involves	0.6%	5 (4–6)	2%	31%
Check you understood the explanation he/she gave for your symptoms	0.6%	5 (4–6)	2%	29%

^a^The theoretical range of scores on single items are 0 to 6. Medians with Inter Quartile Ranges (IQR) are calculated after imputation of missing values

### Distributions of CRQ scores

**Fig. 2:**
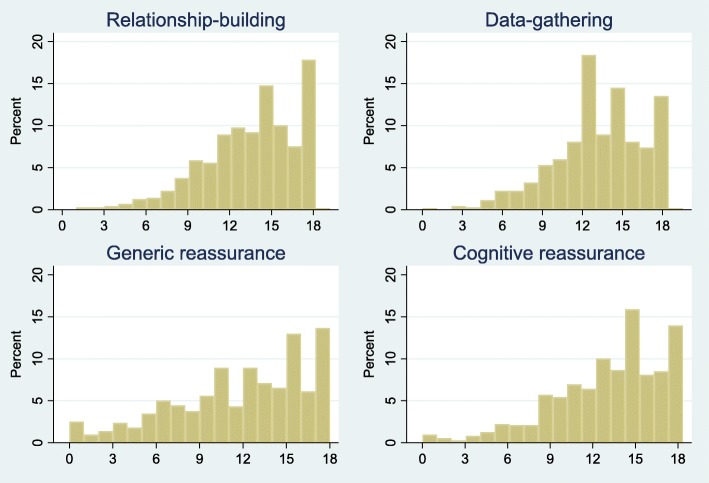
Distribution of scores on the four sub-scales of the Consultation-based Reassurance Questionnaire

All individual CRQ items showed a left-skewed distribution with the proportion of patients scoring the highest possible value ranging from 14 to 49% across items (Table 2). This resulted in left-skewed distributions on all four subscales (Fig. 2). The items that most patients gave a minimum score were *Tell you that you should not be worried* (6%), *Encourage you to voice your concerns regarding your symptoms* (7%) and *Tell you that everything would be fine* (10%). The lowest median value on subscales was observed for generic reassurance (12 IQR [8–15]) and the highest for relationship-building (15 IQR [12–17]) (Table 1).

### Associations with patient characteristics

Associations between CRQ-scores and baseline characteristics were generally weak (Table 3). Scores were slightly lower for patients who had visited a chiropractor previously as compared to those who had not. Other than that, the only statistically significant associations were demonstrated for generic reassurance indicating slightly lower levels of generic reassurance for patients above 50 years, those with the highest level of education, and those with more than 3 months symptom duration, who had 1.9 (95% CI 1.0 to 2.7) points lower scores on generic reassurance than patients presenting with LBP lasting less than 1 month.

**Table 3:** Associations between patient characteristics and the Consultation Reassurance Questionnaire (*n* = 717)

	Data-gathering, β (95% CI)	Relationship-building, β (95% CI)	Generic reassurance, β (95% CI)	Cognitive reassurance, β (95% CI)
Age			*	
< 35 (ref)				
35–50	-0.09 (-0.77; 0.58)	-0.30 (-0.93; 0.33)	-0.13 (-1.01; 0.76)	-0.48 (-1.21; 0.24)
> 50	0.10 (-0.57; 0.77)	-0.14 (-0.76; 0.49)	-1.17 (-2.05; -0.28)	-0.29 (-1.01; 0.43)
Females vs. males	0.32 (-0.19; 0.83)	0.51 (0.03; 0.98)*	-0.27 (-0.95; 0.40)	0.13 (-0.41;0.68)
Education			*	
No qualification (ref)				
Education < 3 years	0.32 (-0.42; 1.07)	0.06 (-0.64; 0.76)	-0.29 (-1.27;0.70)	0.31 (-0.50; 1.11)
Education ≥ 3 years	-0.31(-1.07; 0.46)	-0.35 (-1.07;0.36)	-1.26 (-2.27; -0.25)	-0.33(-0.15; 0.49)
Symptom duration			*	
< 1 month (ref)				
1–3 months	0.69 (-0.11; 1.48)	0.54 (-0.20; 1.28)	-0.06 (-1.10; 0.97)	0.39 (-0.46; 1.25)
> 3 months	0.14 (-0.52; 0.80)	-0.22 (-0.84; 0.39)	-1.86 (-2.72; -1.0)	-0.06 (-0.77; 0.65)
Previous chiropractic care (yes vs. no)	-0.46 (-1.00; 0.05)	-0.60 (-1.07; -0.13)*	-1.19 (-1.85; -0.53)*	-0.56 (-1.01; -0.02)*

*CI* Confidence interval

* Statistically significant association (*p* < 0.05) between the baseline factor and the CRQ sub-scale

### Associations with 2-weeks outcome

**Fig. 3:**
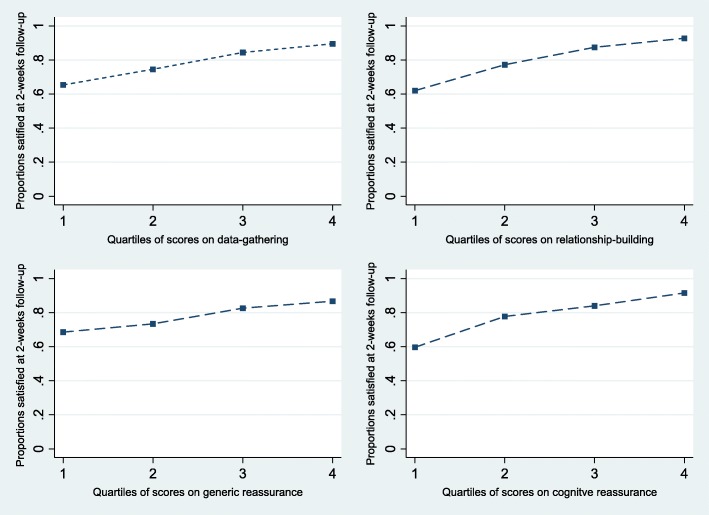
Satisfaction with care in relation to the four subscales of the Consultation-based Reassurance Questionnaire. Proportion of patients in quartiles of baseline scores on each subscale of the Consultation Reassurance Questionnaire that were satisfied with care at 2-weeks follow up

Overall, 77% of the patients were satisfied with care. All subscales of the CRQ were positively associated with patients’ satisfaction with care (Table 4 and Fig. 3), which was not modified by previous chiropractic care (results not reported). The associations were of similar magnitudes across the subscales relationship-building, data-gathering, and cognitive reassurance (Odds ratios 1.18–1.23) and somewhat weaker for generic reassurance (OR = 1.08).

**Table 4:** Associations between the CRQ subscales and 2-weeks outcomes

	Satisfied OR (95% CI) *n* = 623	Pain Control^l#^ β (95% CI) *n* = 623
Data-gathering	1.18 (1.12 to 1.25)*	0.04 (−0.02 to 0.09)
Relationship-building	1.23 (1.16 to 1.31)*	0.06 (−0.001 to 0.11)
Generic reassurance	1.08 (1.04 to 1.13)*	0.07 (0.03 to 0.11)*
Cognitive reassurance	1.18 (1.12 to 1.24)*	0.03 (−0.02 to 0.08)

*OR* Odds Ratio, *CI* Confidence Interval

^#^ adjusted for baseline values of pain control; * *p* < .05

The CRQ was not associated with pain control at 2-weeks follow up except for a weak association with generic reassurance (β = 0.07 [95% CI 0.03–0.11]). There were no statistically significant interactions between previous chiropractic care and reassurance scores for any of the subscales.

## Discussion

This is the first study to test the feasibility of the newly developed CRQ questionnaire outside of the development setting. In a cohort of chiropractic patients with LBP, very few missing values were recorded and the internal consistency of the subscales supported the four domains of the questionnaire. The scores were distributed across the scale with some skewness towards the higher scores on all four subscales. The rather high frequency of the highest possible scores, known as ceiling effect, might be problematic if the scale is considered for use as an outcome measure for educating clinicians in delivering reassuring information. However, for all scales, except relationship-building, the ceiling did not exceed 15% which is considered acceptable [15].

The results suggest that patients generally perceive high levels of reassurance when consulting a chiropractor for LBP with median scores that resembled what has been observed in UK general practice [11]. Low scores were more frequently observed for generic reassurance than for the other subscales, with lower scores associated with longer duration of LBP, higher education and older age. The CRQ was originally tested in a patient population consulting for acute episodes of LBP [10], and it is unknown if high levels of generic reassurance (such as “everything will be fine”, “don’t worry”) should be aimed for also in patients with long-lasting symptoms for whom substantial pain improvement may not be considered realistic. Scores were slightly lower for patients who had seen a chiropractor previously than for those who had not. It may be that these patients did not have much need for information because the clinician has (or believes that she has) provided that information at previous consultations, but it needs to be studied if that is so.

All subscales of reassurance were positively associated with satisfaction with care reported after two weeks. This implies that the CRQ captures elements of care that are of importance to patients, but this study did not investigate if there is a causal relationship between reassurance in the consultation and the higher levels of satisfaction observed. This relationship should be explored in more depth. The CRQ was not associated with pain control at follow up except for a weak positive association with generic reassurance. It may be that other aspects of reassurance actually affect perceived pain control too, but this could not be demonstrated in this sample because of very few low scores on other subscales. Alternatively, the CRQ might not cover aspects of reassurance of importance for perceived pain control, or reassuring information is not sufficient to obtain the skills required to obtain a sense of control.

This study was based on a sample that was adequately sized for exploring the potential relationships between CRQ scores and patient characteristics, and for obtaining sufficiently certain estimates of associations with outcomes. Also, it is clearly a strength of the study that patients received the CRQ on the same day as they had consulted for LBP which reduced the risk of recall bias. Patients were recruited by 18 chiropractors in four clinics and it is unknown if these are representative of Danish chiropractors and to what extent patients’ perceptions of reassurance would be similar in chiropractic practice generally. Approximately 25% of patients consenting to the study did not complete the full baseline questionnaire. These non-responders did differ from the rest of the sample on some parameters, but we find no reason to believe that the relationships between CRQ and other investigated factors and outcomes would be different in those than in the study sample. We included patients with different LBP durations to be able to investigate the potential relationship between duration and perceived reassurance. However, 70% of the study sample reported LBP of less than 4 weeks duration which fits with the population of people with acute LBP that participated in the development of the CRQ.

## Conclusions

The CRQ was feasible for use in a Danish chiropractic setting and scores on all four reassurance subscales related positively to patients’ satisfaction. Generic reassurance was related to perceived pain control after two weeks, and it should be explored further if generic reassurance supports patients in developing a sense of pain control. Since we did not observe very low scores on other aspects of reassurance this study cannot tell to what extent other subscales might influence sense of pain control. Patients who had visited a chiropractor previously reported slightly lower levels of reassuring information, and it should be explored if this is in accordance with patients’ needs. In summary, the CRQ is relevant for studying reassurance in chiropractic settings and there is a need for investigating the impact of reassurance on additional patient outcomes and at later follow up time points.

## Appendix

**Table 5:** Consultation-based Reassurance Questionnaire - Danish

De følgende spørgsmål handler om, hvordan din kiropraktor kommunikerede og optrådte i løbet af konsultationen. Besvar venligst alle spørgsmålene så ærligt som muligt ud fra din hukommelse.								
I hvor høj grad gjorde kiropraktoren følgende …		Slet ikke					I høj grad	
1	Fortalte dig, at alt vil blive godt	o	o	o	o	o	o	o
2	Viste, at han/hun forstod dine bekymringer	o	o	o	o	o	o	o
3	Forsikrede dig, at han/hun ikke var. alvorligt bekymret for din ryg	o	o	o	o	o	o	o
4	Forklarede hvordan den tilbudte behandling ville hjælpe på dit problem	o	o	o	o	o	o	o
5	Fortalte dig, at du ikke skulle være bekymret	o	o	o	o	o	o	o
6	Lyttede opmærksomt, mens du talte	o	o	o	o	o	o	o
7	Sikrede sig, at du forstod hvad din behandlingsplan indebærer	o	o	o	o	o	o	o
8	Beroligede dig	o	o	o	o	o	o	o
9	Opsummerede hvad du havde fortalt	o	o	o	o	o	o	o
10	Viste ægte interesse for dit problem	o	o	o	o	o	o	o
11	Opfordrede dig til at fortælle om dine bekymringer omkring dine symptomer	o	o	o	o	o	o	o
12	Sikrede sig, at du forstod den forklaring han/hun gav dig på dine symptomer	o	o	o	o	o	o	o

## Abbreviations

ChiCo: The (Danish) Chiropractic Cohort
CI: Confidence interval
CRQ: Consultation-based Reassurance Questionnaire
IQR: Inter quartile range
LBP: Low back pain
OPEN: Odense Patient data Explorative Network
